# Decitabine Treatment Induces a Viral Mimicry Response in Cervical Cancer Cells and Further Sensitizes Cells to Chemotherapy

**DOI:** 10.3390/ijms232214042

**Published:** 2022-11-14

**Authors:** Alexia Alexandraki, Katerina Strati

**Affiliations:** Department of Biological Sciences, University of Cyprus, 1 University Avenue, Nicosia 2109, Cyprus

**Keywords:** decitabine, cervical cancer, viral mimicry response, immunomodulation, chemosensitivity, cisplatin, cell viability

## Abstract

Purpose: To investigate the anti-cancer, chemosensitizing and/or immunomodulating effects of decitabine (DAC) to be used as a potential therapeutic agent for the treatment of cervical cancer (CC). Methods: Cervical cancer cell lines were treated with low doses of DAC treatment used as a single agent or in combination with chemotherapy. End-point in vitro assays were developed as indicators of the anti-cancer and/or immunomodulating effects of DAC treatment in CC cells. These assays include cell viability, cell cycle analysis, apoptosis, induction of a viral-mimicry response pathway, expression of MHC-class I and PD-L1 and chemosensitivity. Results: High and low doses of DAC treatment induced reduction in cell viability in HeLa (HPV18+), CaSki (HPV16+) and C33A (HPV−) cells. Specifically, a time-dependent reduction in cell viability of HeLa and CaSki cells was observed accompanied by robust cell cycle arrest at G2/M phase and alterations in the cell cycle distribution. Decrease in cell viability was also observed in a non-transformed immortal keratinocyte (HaCat) suggesting a non-cancer specific target effect. DAC treatment also triggered a viral mimicry response through long-term induction of cytoplasmic double-stranded RNA (dsRNA) and activation of downstream IFN-related genes in both HPV+ and HPV− cells. In addition, DAC treatment increased the number of CC cells expressing MHC-class I and PD-L1. Furthermore, DAC significantly increased the proportion of early and late apoptotic CC cells quantified using FACS. Our combination treatments showed that low dose DAC treatment sensitizes cells to chemotherapy. Conclusions: Low doses of DAC treatment promotes robust induction of a viral mimicry response, immunomodulating and chemosensitizing effects in CC, indicating its promising therapeutic role in CC in vitro.

## 1. Introduction

Cervical cancer (CC) is the fourth most common cancer in females worldwide accounting for 5% of all human cancers (>500,000 cases) per year [1,2]. Persistent infection with high-risk human papillomavirus (HPV) is the causative trigger for the initiation and development in 99% of CC cases [3]. Approximately 570,000 cases and 311,000 deaths from CC cases occurred in 2018 worldwide [1], and it is estimated to cause more than 470,000 deaths by 2030 due to lack of widespread uptake of HPV vaccines [4]. Even though effective HPV-based cervical screening is available, management of early diagnosed HPV+ individuals is limited to monitoring (cervical screening), whilst treatment options become available only after formation of precancerous cervical lesions or CC development [5]. Most cases of CC are diagnosed at the metastatic stages and treatment options include neoadjuvant chemotherapy and/or radiotherapy [6]. Unfortunately, 50% of tumour recurrence occurs within one year post “effective” treatment [7]. Prognosis of patients with tumour recurrence is poor with 5-year survival rate of 5% [7,8,9]. Consequently, introducing new therapeutic approaches for patients with cervical carcinomas is urgently needed.

Epigenetic therapy using DNA methyltransferase (DNMT) inhibitors, such as 5-aza-2′-deoxycytidine (decitabine; DAC), have demonstrated promising therapeutic potential against solid tumours, including CC, in preclinical models and in clinical trials [10,11,12,13,14,15]. DAC has been FDA approved and is currently used for the treatment of haematological malignancies [16]. Treatment with DNMT inhibitors including DAC was previously shown to upregulate crucial genes such as tumour suppressor genes [17,18,19], induce a viral mimicry response paving the way to immunotherapy [20,21,22,23], promote chemosensitivity [24,25,26] and reduce the colony-forming ability in cancer such as colorectal cancer (CRC) [20].

Early findings, focusing on HPV+ CC, showed that DAC treatment depletes the two main HPV oncoproteins E6 and E7 and restores their respective targets p53 and Rb in CC cell lines [27]. It was also demonstrated that DAC treatment re-activates the tumour suppressive miRNA-375, which is correlated with a reduction in the levels of E6 and E7 oncoproteins in CC cells [27]. In addition, the ability of HPV oncoproteins E6 and E7 to manipulate the epigenetic modifications in the host cell to promote carcinogenesis is well recognized [2,28,29,30]. Consequently, targeting aberrant DNA methylation by DNMT inhibitors may have a therapeutic potential in cervical carcinogenesis.

The anti-cancer potential of DNMT inhibitors lies beyond the reactivation of epigenetically silenced genes. The interest has now shifted to the potential of using DNMT inhibitors as immunomodulators and/or chemosensitizers [17,20,31,32,33,34]. It has been shown by others that transient, low dose treatment of DNMT inhibitors, including DAC, induce a “viral mimicry” response, which is partially initiated through activation of endogenous retroviral elements (ERVs) [20,21]. ERVs form double-stranded RNA (dsRNA) structures in the cytoplasm, which are then recognized by a pattern recognition receptor, the melanoma differentiation-associated gene 5 (MDA5), followed by activation of an interferon (IFN) immune response in cancer cells including CRC cells [20,21].

Activation of the IFN-response pathway by DNMT inhibitors could increase tumour recognition by the immune system and tumour susceptibility to immunotherapy (i.e., immune check-point inhibitors) or chemotherapy [35,36]. Yu et al., 2019 [31], demonstrated that low-dose DAC treatment enhances the anti-tumour effect of the PD-1 blockade, triggers upregulation of immune-related genes, such as major histocompatibility complex genes, and stimulates T-cell infiltration in CT26 CRC-bearing mouse models. In addition, it was previously shown by others that DAC can improve tumour immunogenicity by inducing upregulation of various immune-related molecules, including the major histocompatibility complex class I (MHC-I) and the natural-killer group 2, member D (NKG2D), indicating the immunomodulating capacity of DAC in solid tumours [36]. However, the anti-cancer and/or immunomodulating potential of DNMT inhibitors, including DAC, in CC remains to be further elucidated. Cisplatin, a platinum-based drug, is currently used as a standard chemotherapy for the treatment of advanced CC [37,38]. However, resistance to cisplatin may occur, hence compromising its use for the treatment of advanced or recurrent CC [39,40]. Preclinical and clinical studies showed that DAC treatment demonstrated a promising therapeutic efficacy when tested in combination with platinum-based drugs (e.g., cisplatin) in patients with advanced CC and other cancers [11,41,42,43,44]. However, the role of DAC treatment in enhancing the response of CC to chemotherapy remains to be further investigated. The aim of this study is to assess the anti-cancer and/or immunomodulating potential of DAC treatment in CC cells and also to determine whether DAC treatment facilitates an enhanced response of CC cells to chemotherapy.

## 2. Results

### 2.1. Changes in Cell Viability of CC Cell Lines in Response to DAC Treatment

To determine changes in cell viability of CC cell lines following DAC treatment, an MTT assay was performed. HeLa (HPV18), CaSki (HPV16) and C33A (HPV−) cells were treated with a range of DAC concentrations (0.015–32 μΜ) for 72 h. In addition, to assess whether DAC treatment promotes a cancer-specific cytotoxicity, DAC treatment was performed using a non-transformed immortal keratinocyte cell line, HaCat. Briefly, cells were treated with DAC for 72 h (DAC was replenished every 24 h) and cell viability assay was performed 24 h following the last treatment (0-day post-treatment). We have shown that cell viability was reduced in response to the low and high doses of DAC in all CC cells. However, this effect was not dose-dependent at this short time point assessed (Figure S1A–D). For this reason, cell viability of CC cells in response to DAC was assessed at longer time points after DAC removal in accordance with previously published literature [20,21]. In addition, HaCat cells showed significant reduction in cell viability at all DAC concentrations compared to the untreated cells (Figure S1D). These results suggest that the effects of DAC treatment are not cancer-specific, an important consideration for its clinical use. For the remainder of this study, low doses of DAC were used to promote the optimum anti-cancer potential of DAC in CC. The selected doses were derived from previously published literature [20,27].

### 2.2. DAC Treatment Activates a Viral Mimicry Response in CC Cells

#### Induction of dsRNA Levels and IFN-Related Genes in Response to DAC Treatment in CC Cells

**Figure 1:**
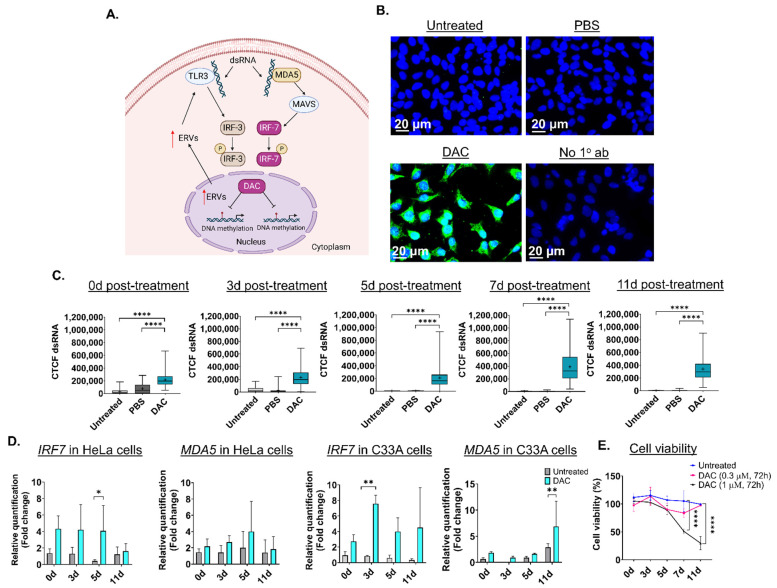
Low dose DAC treatment induces a viral mimicry response in HPV+ cells and HPV− cells in response to DAC treatment. (**A**) Illustration of the viral mimicry response induced by DAC treatment in cancer cells [20]. Figure created in BioRender [20,21,45]. (**B**) ICC/IF analysis of dsRNA in response to DAC (1 μΜ, 72 h) in HeLa cells at 11d post-treatment. Blue staining (DAPI) indicates the nucleus, and green fluorescence signal (AlexaFluor^®^ 488) indicates the staining for dsRNA. Untreated and PBS-treated cells were used as controls. Cells incubated without the primary antibody were used as the negative control. (**C**) Quantification of dsRNA levels in HeLa cells in response to DAC treatment at 0d, 3d, 5d, 7d and 11d was performed by measuring the corrected total cell fluorescence (CTCF) using ImageJ as follows: CTCF = Integrated density − (area of selected cell × mean fluorescence of background readings [20]. Data represent the median with range of CTCF value from 100 cells per treatment condition for each time point. The mean is also indicated by a cross (+). The experiment is a representative repeat of two independent experiments. Statistical analysis was performed using one-way ANOVA with Kruskal–Wallis test (**** *p* < 0.0001). (**D**) QPCR analysis of *IRF7* and *MDA5* gene expression levels in response to DAC treatment (0.3 μΜ, 72 h) at the indicated time points in HeLa cells and C33A cells. For all qPCR analysis, normalization to the mRNA levels of the housekeeping *GAPDH* was conducted. Relative quantification to the vehicle control (PBS-treatment) was performed. The fold change (2^−ΔΔCt^) was calculated and plotted showing the mean ± SD. The data presented are a representative repeat of two independent experiments. Statistical analysis was performed using two-way ANOVA with Tukey’s multiple comparisons test (* *p* < 0.05, ** *p* < 0.01). (**E**) Cell viability of HeLa cells in response to DAC treatment (1 μΜ, 0.3 μM), for 72 h at 0d, 3d, 5d, 7d and 11d post-treatment using an MTT assay. The data are a representative repeat of three independent experiments. The data are plotted as the mean ± SD. Statistical analysis was conducted using two-way ANOVA with Tukey’s multiple comparisons test (**** *p* < 0.0001).

Based on previous research, transient, low-dose DAC treatment (0.3 μΜ, 24 h) induces formation of dsRNA structures in CRC cell lines, partially due to activation of ERVs followed by a “viral mimicry” response [20,21] (Figure 1A). DAC-mediated induction of dsRNA triggers the upregulation of the dsRNA-sensing molecule, *MDA5,* and interferon-related genes, including the *interferon regulatory factor 7* (*IRF7*) (Figure 1A) [20]. To assess whether DAC treatment induces formation of dsRNA in CC cells, HeLa, CaSki and C33A cells were treated with DAC at 1 μΜ for 72 h, and dsRNA levels were assessed at 0 days (0d), 3 days (3d), 5 days (5d), 7 days (7d) and 11 days (11d) post-treatment, as illustrated in Figure S2. Briefly, cells were treated with DAC for 72 h (DAC was replenished every 24 h). DAC was replaced with fresh drug-free media 24 h following the last treatment. Cells were then allowed to grow under drug-free conditions up to 11d post-treatment. ICC/IF analysis showed that DAC treatment (1 μΜ, 72 h) induced dsRNA levels in the cytoplasm of HeLa cells at all time points tested compared to untreated cells and the PBS-treated cells (vehicle control) (Figure 1B and Figure S3A). In addition, induction of dsRNA levels was also noted in the CaSki and C33A cells and in the non-transformed immortal keratinocyte cell line, HaCat cells, at 3d post-treatment, as shown by ICC/IF analysis (Figure S3A).

Quantification of dsRNA levels in HeLa cells using ImageJ was performed showing that DAC treatment results in a statistically significant increase in dsRNA levels at 0d, 3d, 5d, 7d and 11d post-treatment (Figure 1C). A statistically significant increase in dsRNA levels was also noted in CaSki, C33A and HaCat cells (**** *p* < 0.0001) (Figure S3B). To assess whether DAC-mediated induction of dsRNA is followed by downstream activation of the dsRNA sensor molecule, *MDA5*, and interferon-related genes, including the *IRF7* [20], we performed transcriptional analysis using qPCR. QPCR analysis showed that DAC treatment induced mRNA upregulation of *IRF7* and *MDA5* in HeLa cells in response to a lower dose of DAC treatment (0.3 μΜ, 72 h) (Figure 1D).

Specifically, an increase in gene expression of *IRF7* was noted at all time points tested (5d post-treatment, * *p* < 0.05). Gene upregulation of *IRF7* was accompanied by an increase in the *MDA5* mRNA levels in response to low dose DAC treatment in HeLa cells (Figure 1D). Similarly to the HPV+ cell line, an increase in the *IRF7* and *MDA5* levels in response to DAC treatment was also noted in the HPV− cell line, C33A (Figure 1D).

Prolonged induction of viral mimicry response in HeLa cells was associated with progressive decrease in cell viability, with the greatest reduction noted at 11d post-treatment, in response to 1 μΜ, 72 h (Figure 1E). In contrast, non-statistically significant changes in cell viability were noted in response to the nanomolar dose of DAC treatment (Figure 1E). Similarly, induction of dsRNA in CaSki cells was associated with statistically significant time-dependent reduction in cell viability at the indicated time points tested in response to DAC treatment at 1 μΜ, 72 h (Figure S3C).

### 2.3. DAC Treatment Induces Abnormalities in the Cell Cycle Distribution of HeLa Cells

**Figure 2:**
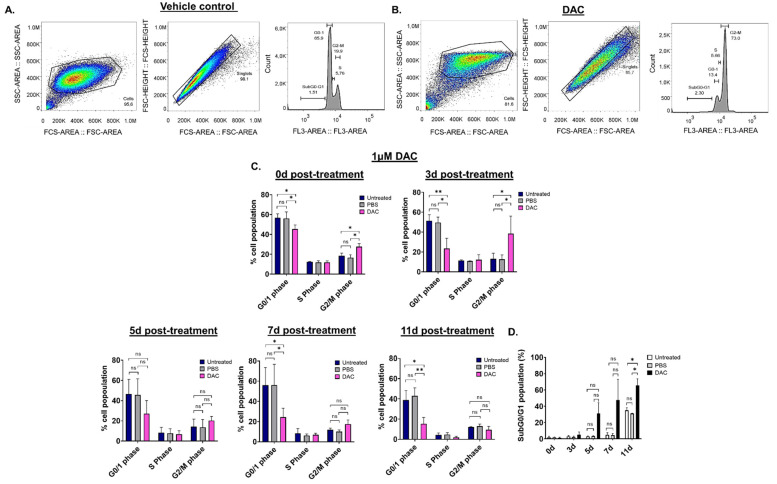
DAC treatment induces changes in cell cycle distribution in HeLa cells. Cell cycle analysis was performed to determine potential changes in the cell cycle of HeLa cells in response to DAC treatment (1 μΜ, 72 h) using flow cytometry. The appropriate gating was conducted to select the desired cell population for the analysis, as shown by the raw data presented at 3d post-treatment for the PBS-treated cells (vehicle control) (**A**) and the DAC-treated cells (**B**). (**C**) The graphs show the mean percentage of cell population for each of the cell-cycle phases (G0/1 phase, S phase, G2/M phase) in response to DAC treatment at 1 μΜ, 72 h at the indicated time points (0d, 3d, 5d, 7d, 11d). (**D**) The percentage of the mean population at the SubG0/G1 was plotted for each of the time-points post-treatment (0d, 3d, 5d, 7d, 11d post-treatment) in response to 1 μΜ DAC treatment (72 h). The data represent average values from three independent experiments. The data are plotted as the mean ± SD. Statistical analysis was performed using two-way ANOVA with Tukey’s multiple comparisons test (* *p* < 0.05, ** *p* < 0.01, ns = not statistically significant).

To assess whether DAC treatment results in any changes in the cell cycle of CC cells, cell cycle distribution analysis was performed using flow cytometry. Since the effects of DAC treatment were similar in all cell lines used, cell cycle analysis was performed in HeLa cells. HeLa cells were treated with 1 μΜ of DAC for 72 h, and cell cycle distribution was assessed at 0d, 3d, 5d, 7d and 11d post-treatment. The nucleus of untreated, PBS-treated (vehicle control) and DAC-treated cells was stained with propidium iodide (PI). Appropriate gating was conducted, and the cell cycle phases were identified and annotated (Figure 2A,B). Our data showed that DAC treatment resulted in changes in the cell cycle distribution partly characterized by accumulation of cells at the G2/M phase in a time-dependent manner (Figure 2C). In particular, cell cycle arrest at the G2/M phase was noted at 0d and 3d post-treatment in response to DAC treatment at 1 μM with the greatest increase in cell population at the G2/M phase observed at 3d post-treatment (3-fold) compared to the vehicle control (Figure 2A–C). Simultaneously, the cells at the G0/1 phase remained low at all times (3d, 5d, 7d, 11d) compared to untreated and the vehicle-treated cells (Figure 2C). Following the cell cycle arrest, most of the DAC-treated cells ended up in the SubG0/G1 phase at 5d, 7d and 11d post-treatment, indicating that the cells may undergo apoptosis (Figure 2D).

### 2.4. DAC Treatment Induces Cellular Apoptosis in HeLa Cells

**Figure 3:**
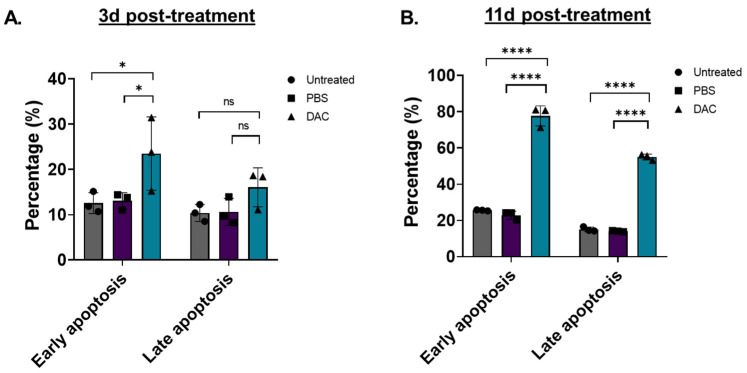
DAC treatment induces early and late apoptosis in HeLa cells. Flow cytometry analysis of HeLa cells stained with Annexin-V (early apoptosis) and 7-AAD (late apoptosis) in response to DAC treatment. Cells were treated with 1 μM DAC, 72 h, and apoptosis was assessed at 3d (**A**) and 11d post-treatment (**B**). The percentage of early and late apoptotic cells is plotted in response to DAC treatment. The data represent average values from three independent experiments (mean ± SD). Statistical analysis was performed using two-way ANOVA with Tukey’s multiple comparisons test (* *p* < 0.05, **** *p* < 0.0001, ns = not statistically significant).

To assess whether DAC-mediated induction of cell viability is associated with cell apoptosis, as suggested by cell cycle analysis, flow cytometry was performed (Figure 3). Two different time points were tested, an early time point at 3d post-treatment and a late time point at 11d post-treatment. To distinguish between early and late apoptosis, HeLa cells were stained with Annexin-V and 7-AAD, respectively, as previously described [46,47,48]. We found that DAC treatment induced a higher number of early and late apoptotic cells at 3d post-treatment by 1.8-fold (* *p* = 0.0254) and 1.5-fold (*p* = 0.2870), respectively, compared to the vehicle control (Figure 3A). A striking effect was observed at 11d post-treatment, where an approximately 3.4-fold and 4-fold increase in early and late apoptotic cells, respectively, was induced in response to DAC treatment compared to the vehicle-control cells (Figure 3B).

### 2.5. Anti-Cancer Effects of Combination Treatment of DAC and Cisplatin in CC Cell Lines

#### 2.5.1. Cell Viability of CC Cells in Response to Cisplatin Alone

Since the use of DAC in solid tumours is more effective as part of a combination treatment, we decided to investigate its combinatorial cytotoxicity in CC cell lines. Initial experiments focused on assessing the cell viability of HeLa, CaSki and C33A cells in response to low and high doses of cisplatin, a platinum-containing chemotherapeutic agent, prior to combination treatments, using an MTT assay (Figure S4A–C). This was performed to identify low doses of cisplatin that exhibit reduction in cell viability but without causing severe cytotoxicity. The range of cisplatin concentrations tested (0.156 μM, 0.3125 μΜ, 0.625 μΜ, 1.25 μΜ, 2.5 μΜ, 5 μΜ, 10 μΜ, 20 μΜ) was selected to include the range of doses used in previously published literature [49]. Cells were treated with cisplatin for 72 h, and cell viability assay was conducted at 4d post-treatment (Figure S4A–C).

Our data showed that the two lowest cisplatin doses (0.156 μΜ, 0.3125 μΜ) resulted in a significant reduction in cell viability by 1.1- and 1.4-fold, respectively, in HeLa cells compared to the untreated cells (* *p* < 0.05) (Figure S4A), but did not cause any significant reduction in cell viability in CaSki cells (Figure S4B). Similarly, low doses of cisplatin (0.156 μM, 0.3125 μΜ, 0.625 μΜ) did not cause any drop in cell viability in the case of C33A cells compared to the untreated cells (Figure S4C). Significant reduction in cell viability of C33A cells was noted in response to the higher doses of cisplatin (1.25 μΜ, 2.5 μΜ, 5 μΜ, 10 μΜ, 20 μΜ). Overall, cell viability followed a dose-dependent reduction in response to the low and high doses of cisplatin in all CC cell lines tested. The greatest reduction in cell viability was observed at 20 μΜ resulting in 411-fold reduction in C33A, 134-fold in CaSki, and 93-fold in HeLa cells (Figure S4A–C).

#### 2.5.2. The Anti-Cancer Effect Induced in Response to Combination Treatment Compared to Each Agent Alone in CC Cell Lines

**Figure 4:**
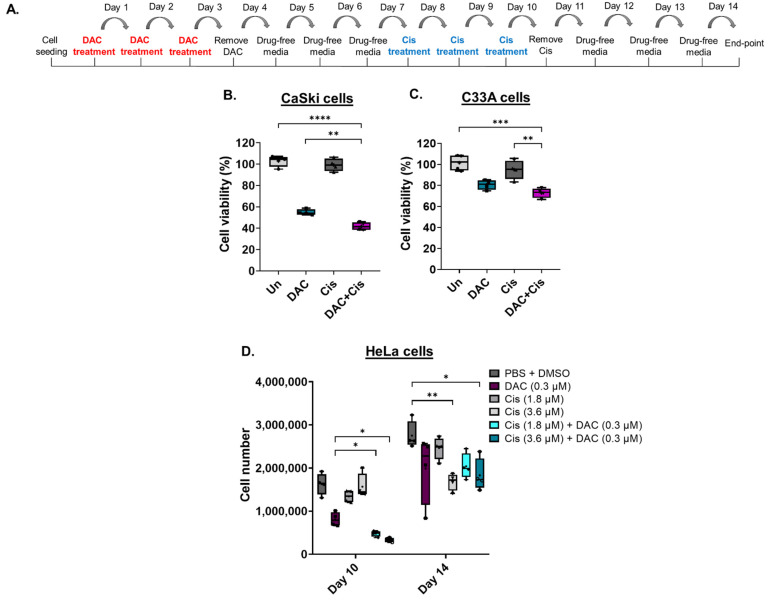
Low-dose DAC treatment sensitizes CC cells to cisplatin. (**A**) Timeline of DAC treatment (coloured in red) at 0.3 μΜ, 72 h followed by treatment with cisplatin (coloured in blue) at 0.6 μM, 72 h. Cells were harvested at 4 days after cisplatin treatment. Cell viability analysis of CaSki (**B**) and C33A (**C**) cells in response to the combination treatment of DAC (0.3 μΜ, 72 h) followed by cisplatin (0.6 μΜ, 72 h). Statistical analysis was performed using one-way ANOVA (Kruskal–Wallis test) (** *p* < 0.01, *** *p* < 0.001, **** *p* < 0.0001). (**D**) HeLa cells were treated with DAC at 0.3 μΜ, 72 h, followed by cisplatin treatment at 1.8 μΜ or 3.6 μΜ for 72 h. Cell numbers were assessed using a cell counter at the indicated time points of treatment. The following treatment groups were included in the experiment: DAC alone (0.3 μΜ, 72 h); cisplatin alone (1.8 μΜ, 72 h or 3.6 μΜ, 72 h); DAC treatment followed by cisplatin at 1.8 μΜ, 72 h or 3.6 μΜ, 72 h; PBS + DMSO treated cells used as the vehicle control. Cell number of adherent HeLa cells of all treatment conditions were counted at day 10 and day 14, as indicated in the timeline (**A**). The data presented are a representative repeat of three independent experiments. The data are plotted as the median with range. The mean is also indicated by a cross (+). Statistical analysis was performed using one-way ANOVA (Kruskal–Wallis test) test (* *p* < 0.05, ** *p* < 0.01).

To assess whether DAC treatment enhances the response of CC cell lines to cisplatin, cells were treated with a low-dose DAC treatment (0.3 μΜ, 72 h) followed by a low dose of cisplatin (0.6 μΜ, 72 h) (Figure 4A). Specifically, CaSki and C33A cells were treated with low dose DAC for 72 h. Culture medium was removed, and the cells were allowed to grow under drug-free conditions up to 4d post-treatment when cisplatin was added for 72 h. Culture medium was removed and replaced with fresh medium without drug. Cells were allowed to grow under drug-free conditions and harvested at 4 days after cisplatin treatment for cell viability analysis using MTT assay, as illustrated (Figure 4A). We found that pre-treatment of cells with DAC followed by cisplatin treatment significantly reduced cell viability compared to DAC alone (** *p* < 0.01) in CaSki cells (Figure 4B). A significant decrease in cell viability in response to combination treatment was also noted in C33A cells compared to cisplatin alone (** *p* < 0.01) and DAC alone (Figure 4C). Specifically, cell viability was reduced by approximately 13% and 8% in CaSki and C33A cells, respectively, in response to combination treatment, compared to DAC alone.

To examine whether low-dose DAC treatment increases the response of HeLa cells to cisplatin, combination treatments were conducted, and cell numbers were measured using an automatic cell counter. Combination treatment was performed as described in Section 4.6. Specifically, cells were treated with low-dose DAC treatment (0.3 μM, 72 h) and allowed to grow in drug-free medium up to 4d post-treatment. HeLa cells were then treated with cisplatin (1.8 μM or 3.6 μM, 72 h). Cell counting was performed upon completion of cisplatin treatment at day 10 and at day 14, as illustrated (Figure 4A). Based on our analysis, we showed a significantly reduced cell number of adherent HeLa cells in response to DAC treatment followed by cisplatin treatment at either 1.8 μM or 3.6 μM compared to DAC alone (* *p* < 0.05) or cisplatin alone at day 10 (Figure 4D). No statistically significant differences were noted when DAC treatment was followed by cisplatin at 1.8 μM compared to DAC treatment followed by cisplatin at 3.6 μM, even though for the latter there was a trend towards higher cell number reduction.

Cell counting at a later time point (day 14) showed no statistically significant differences in the cell number of HeLa cells in response to combination treatment compared to DAC alone or cisplatin alone (Figure 4D). However, the cell number of cells treated with each agent alone or combination treatment remained lower compared to the vehicle control (PBS + DMSO), with statistically significant differences noted in the cells treated with cisplatin alone at 3.6 μM and in the cells treated with DAC followed by cisplatin treatment at 3.6 μM (Figure 4D).

In addition to the adherent cells, we were also interested in monitoring any changes observed in the number of non-adherent cells in the supernatant. To assess this, non-adherent cells were collected after DAC treatment followed by cisplatin. Cells were collected at day 8, day 9 and day 10, as illustrated in Figure S5A. Our results showed a trend towards a higher number of non-adherent cells in the combination treatment group that received DAC treatment (0.3 μΜ, 72 h) followed by cisplatin (Day 8) (Figure S5B), with a more potent effect on day 9 showing statistical significance (* *p* < 0.05) (Figure S5C). The same trend, but at a lesser degree, was noted at day 10 of cisplatin treatment (Figure S5D). Overall, our results suggest that low-dose DAC treatment enhances the response of HeLa cells to cisplatin treatment, and this effect is more obvious at the earlier time points after cisplatin treatment of DAC pre-treated cells.

### 2.6. Induction of MHC-Class I and PD-L1 in Response to DAC Treatment in CC Cell Lines

**Figure 5:**
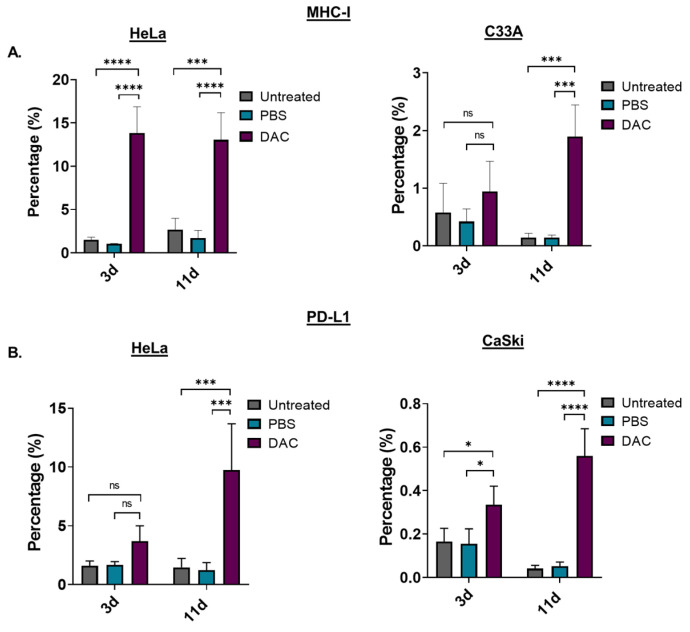
DAC treatment induces MHC-class I and PD-L1 expression on CC cells. (**A**) Percentage of the MHC-I positive cells in the untreated, PBS-treated and DAC-treated HeLa and C33A cells at 3d and 11d post-treatment. (**Β**) Cell numbers of PD-L1 positive cells in untreated, PBS-treated and DAC-treated HeLa and CaSki cells at 3d and 11d post-treatment. The cell number was normalized to the counting beads for each condition. The data represent average values from three independent experiments (mean ± SD). Statistical analysis was performed using two-way ANOVA with Tukey’s multiple comparisons test (* *p* < 0.05, *** *p* < 0.001, **** *p* < 0.0001, ns = not statistically significant).

To assess the immunomodulating effects of DAC treatment, the levels of the antigen-presenting molecule, the major histocompatibility complex I (MHC-I), in response to treatment was examined using FACS analysis. HeLa and C33A cells were treated with DAC at 1 μΜ, 72 h, and cells were analysed based on the MHC-I staining at two different time points: 3d post-treatment and 11d post-treatment. Our FACS analysis data showed that DAC treatment significantly increased the number of cells expressing MHC-I in HeLa cells at both 3d and 11d post-treatment (**** *p* < 0.0001). In addition, a significantly higher percentage of cell population expressing MHC-I was noted at 11d post-treatment (*** *p* = 0.0003) compared to the 3d post-treatment in the case of C33A cells (Figure 5A). In addition, we showed that DAC treatment resulted in increased numbers of HeLa and CaSki cells expressing PD-L1 at 3d post-treatment compared to PBS-treated and untreated cells (Figure 5B). Even greater increase in PD-L1 expressing cells was noted at the later time point, 11d post-treatment, compared to the 3d post-treatment in both HeLa (*** *p* = 0.0002) and CaSki cells (**** *p* < 0.0001).

## 3. Discussion

This study showed that DAC treatment exhibits chemosensitizing and/or immunomodulating effects in CC in vitro. In our study, we assessed the anti-cancer, chemosensitizing and immunomodulating capacity of low-dose DAC treatment as determined by the end-point in vitro assays developed.

We found that DAC treatment induced reduction in cell viability of HPV+ and HPV− CC cell lines using a range of low and high DAC doses. However, there was not a dose-dependent effect in all cell lines tested at the short time point assessed (72 h). It has been previously reported that even though DAC has cytotoxic effects at high doses [50], at lower dose ranges, the effect of DAC is mediated through reactivation of various transcripts and lags in terms of timeline [20]. This might also be explained by the new mechanism of action of DAC to induce a viral mimicry response in cancer cells [20]. This mechanism of action, which is suggested to underly its clinical efficacy, can explain the prolonged time to response to treatment observed in patients [20]. Indeed, in our study, a dose-specific effect was only evident at the later time point post-treatment. Specifically, treatment of CC cells with 1 μΜ DAC resulted in a prolonged reduction in cell viability of CC cell lines following DAC removal. This was sustained up to later time points post-treatment, showing even greater reduction in cell viability compared to earlier time points. These data may present a therapeutic window for secondary anti-cancer therapies. Our data also suggest that DAC-mediated reduction in cell viability occurs in a non-cancer specific manner as indicated by the reduction in cell viability of a non-transformed immortal keratinocyte (HaCat). This was accompanied by increased levels of dsRNA in this cell line.

Our data further support that use of DAC can be a “double-edged sword”, as previously characterized by others [51], as it can result in off-target effects. For this reason, DAC is preferentially used at low doses, in the clinical setting, which favours its epigenetic effect rather than its cytotoxic potential [13,15]. Our data and others [52] suggest that careful monitoring of patients receiving DNA demethylating agents is required. Efforts towards enhancing the efficacy of DAC include the targeted delivery of DAC-loaded nanoparticles [53,54], which can improve the stability of DAC [55], prevent DAC degradation by cytidine deaminase in the liver, allow use of low doses of DAC and reduce off-target effects [56].

Low doses of DAC treatment were assessed in this study, selected based on previously published literature [17,20,27,31]. In particular, the nanomolar dose of 0.3 μΜ that was tested in this study in vitro, mimics the low but effective doses of DAC (e.g., 10 mg/m^2^–30 mg/m^2^) used clinically and that are associated with minimal toxicity [13,15,57]. Our data showed that DAC treatment resulted in a prolonged induction of cytoplasmic dsRNA in HPV+ CC cell lines. Such an effect was evident in the HPV− CC cell line. Following induction of dsRNA, upregulation of the dsRNA-sensing molecule, *MDA5*, and the IFN-related gene, *IRF7*, was noted in both HPV+ and HPV− cell lines in response to DAC treatment. These results suggest that low dose DAC treatment induce a “viral mimicry” response in HPV+ and HPV− cell lines. This response was previously identified in other cancer types, including CRC, but has not been previously described in CC [20,21]. These data suggest that the ability of DAC to induce an anti-viral response in CC cells may pave the way to novel therapeutic approaches against CC, including combination treatment with immune checkpoint therapies (i.e., anti-CTL4) [21,58].

We have also found that DAC treatment at 1 μΜ results in changes in cell cycle distribution, partly characterized by cell cycle arrest at the G2/M phase in HPV+ cell line, HeLa. Cell cycle arrest was observed at the earlier time points post-treatment (0d, 3d) followed by increased cell population in the subG0/G1 phase at the later time points post-treatment (5d, 7d, 11d) in response to 1 μM DAC treatment. Cell cycle arrest at G2/M phase was also reported in other cancer types, such as gastric cancer and lung carcinomas, and it was reported to occur in a p53-independent manner [49].

Increase in the subG0/G1 population may indicate induction of apoptosis. Indeed, we showed that DAC treatment results in early and late apoptosis at 3d post-treatment, and this effect is tremendously increased at a later time point (11d post-treatment). These results agree with the cell cycle analysis performed (SubG0/G1). We have shown that DAC treatment mediates apoptosis in CC cells; however, the pathway through which apoptosis is induced in CC cells has not been identified in this study. Previously published data showed that DAC treatment induces a p53-independent apoptosis through reactive oxygen species and caspase activation [59]. Future experiments to assess the mechanism by which DAC treatment induces disruption in the cell cycle distribution followed by apoptosis in CC cells will further enhance the understanding of how DAC treatment promotes its anti-cancer effect in these cell lines.

Despite the fact that DAC is currently used as a monotherapy for the treatment of haematological cancers [16], clinical trials showed that DAC alone has not been effective for the treatment of solid tumours [60,61]. DAC treatment exhibits its optimum anti-cancer potential when is used in combination with other anti-cancer drugs [15,62] or immunotherapy [31].

This is primarily because DAC treatment sensitizes cancer cells to chemotherapy [25], enhances tumour immunogenicity [22,23,31,63] and increases response to immunotherapy including immune checkpoint inhibitors [31]. The best therapeutic outcome of epigenetic drugs including DAC was shown to be the use of low but effective doses that favour epigenetic tumour reprogramming, which can stimulate the anti-cancer effects of other treatments [13,17,19,31,41,44]. Previous data showed that DAC treatment sensitizes cancer cells to chemotherapy, allowing low doses of chemotherapy to be used, which in turn reduces toxicity [13,15]. Cisplatin, a platinum-based drug, is currently used in clinical practice for the treatment of advanced CC; however, tumour recurrence occurs in 40% of patients [37,38,64]. In addition, tumour resistance to cisplatin often compromises its efficacy to treat advanced or recurrent CC, resulting in poor survival [10,40,41]. Consequently, additional therapeutic approaches are required to improve the therapeutic outcome in these patients. Ramakrishnan et al., 2017 [65], showed that DAC treatment sensitizes osteosarcoma cells to cisplatin via activation of NOTCH1 signaling [65]. Epigenetic reprogramming by DAC treatment resulting in reactivation of the DNA mismatch repair gene, *MLH1*, and pro-apoptotic genes (e.g., *apoptosis protease-activating factor 1*, *Apaf1*) was shown to be associated with reverse chemoresistance to platinum drugs such as carboplatin or cisplatin. In this study, we showed that low-dose DAC treatment significantly enhances the response of CC cells to low dose cisplatin in vitro. We also identified endpoints at which the greatest anti-cancer effect of the combination treatment is observed, as measured by an automatic cell counting or the MTT assay. The greatest effect of the combination treatment was observed following early time points after addition of cisplatin to pre-treated cells with DAC, whilst cell recovery is noted at later time points (Day 14). Our findings suggest that a combination regimen of DAC treatment followed by cisplatin treatment could be potentially used as a therapeutic approach in CC. Future experiments will aim to uncover the mechanisms through which DAC treatment induces chemosensitivity to cisplatin in CC cells using transcriptional and functionality assays.

In addition, we found a prolonged induction of the antigen-presenting molecule, MHC-class I, in HPV+ and HPV− cells in response to DAC treatment. It is hypothesized that HPV targets tissue stem cells facilitating immune evasion and persistent HPV infection resulting in carcinogenesis [66,67]. Downregulation of MHC-I could facilitate HPV-infected cells to evade the adaptive immune response [68]. Consequently, the ability of DAC treatment to upregulate MHC-I in HPV+ cell lines may indicate the promising potential of DAC to promote immune recognition of HPV+ tumours. We have also shown an increase in the number of HPV+ CC expressing PD-L1. Expression of PD-L1 in tumour cells could assist cancer cells to avoid recognition by the T-cells facilitating immune escape [69,70]. However, further induction of PD-L1 in CC cells following treatment with low dose DAC could enhance response to PD-1 blockade, which can increase tumour visibility to the immune system. Similarly, previous studies have demonstrated that DAC remodels the tumour microenvironment promoting T-cell tumour infiltration, triggering the expression of PD-L1 and enhances the response to PD-1 blockade in CRC mouse models [31,64]. While our results indicate statistically significant changes, further studies are needed in order to verify whether DAC-mediated induction of PD-L1 in CC cells, as shown in our study, is clinically significant. In addition, our results indicate that DAC treatment promotes anti-cancer and immunomodulating effects in CC cells independent of the HPV-status of CC cells, suggesting that these findings may be applied to other cancer types that are not virally mediated cancers and potentially to other HPV-associated malignancies including head and neck squamous cell carcinomas (HNSCC), which affect both sexes.

## 4. Materials and Methods

### 4.1. Cell Lines and Cell Culture

The human cervical cancer cell lines, HeLa (HPV18+), were purchased from the Chemoscience Life Science Solutions (CLS, Eppelheim, Germany), whilst CaSki (HPV16+) and C33A (HPV−) CC cells were purchased from the American Type Culture Collection (ATCC, Manassas, VA, USA). CaSki cells were cultured in RPMI 1640 medium, whereas HeLa and C33A cells were maintained in DMEM and MEM (1% L-glutamine), respectively. HaCat cells were purchased from CLS and cultured in DMEM. All cell culture media were supplemented with 10% (*v*/*v*) heat-inactivated foetal bovine serum (FBS) (Sigma-Aldrich, Dorset, UK) in the absence of antibiotics. Cells were incubated in a 5% CO_2_ humidified incubator at 37 °C.

### 4.2. Drug Treatments

DAC (5 mg) (Sigma-Aldrich, A3656, Dorset, UK) was dissolved in PBS (1×) to prepare a stock of 15 mM. Cisplatin (100 mg) (European Pharmacopoeia, Darmstadt, Germany, C2210000) was dissolved in dimethyl sulfoxide (DMSO, Sigma-Aldrich, Dorset, UK) to prepare a stock of 30 mM. Cisplatin stock was filtered using 0.2 μΜ filters (Corning, Berlin, Germany) before use. HeLa, CaSki and C33A cells were seeded in six-well plates at a density 4 × 10^5^, 2.5 × 10^5^, 3 × 10^5^ cells/well, respectively. Following 24 h of cell seeding, cells were treated with DAC at 1 μM or 0.3 μM for 72 h. Media was replaced with fresh media containing DAC every 24 h. Untreated and PBS-treated cells were used as controls. Following 72 h treatment, cells were allowed to grow under drug-free conditions up to the point of harvesting. ICC/IF analysis and RNA extraction were performed at 0 days (0d), 3 days (3d), 5 days (5d), 7 days (7d) and 11 days (11d) post-treatment. Figure 1A, showing the mechanism of action of DAC treatment, was created in Biorender.com. For cisplatin treatment, HeLa, CaSki and C33A cells were seeded in 96-well plates at 0.5 × 10^5^ cells/well. Cells were treated with cisplatin 24 h after cell seeding. For combination treatments, HeLa cells were treated with DAC at 0.3 μΜ for 72 h and media was replaced with fresh media containing DAC every 24 h. Cells were allowed to grow under drug-free conditions. At 3d post-treatment, cisplatin was added every 24 h for 72 h. Cells were allowed to grow under drug-free conditions up to the endpoint.

### 4.3. Reverse-Transcription PCR

Total cellular RNA extraction was performed based on the TRIzoL protocol. The extracted RNA was then DNase-treated using TURBO DNA-free^TM^ kit (Ambion, Life Technologies, Waltham, MA, USA). RNA concentration was quantified using the nanodrop, and 1 μg/ μL of RNA was used for first-strand cDNA synthesis. cDNA synthesis was performed using the 1st stand cDNA Synthesis Kit (PrimeScript, Takara, Saint-Germain-en-Laye, France) with random hexamer primers, according to the manufacturer’s guidelines. The cDNA samples were kept at −20 °C. Reverse transcription polymerase chain reaction (RT-PCR) was performed using the KAPA Taq PCR kit (Kapa Biosystems, Darmstadt, Germany). All primer sequences for performing qPCR analysis were designed to cross an intron-exon boundary. The primer sequences were self-designed using the NCBI primer design tool. The primer sequences are as follows: *IRF7* (FOR): 5′-CCCCACGCTATACCATCTAC-3′; *IRF7* (REV): 5′-CAGCTCCATAAGGAAGCAC-3′; *MDA5* (FOR): 5′-CACTTCCTTCTGCCAAACTTG-3′; *MDA5* (REV): 5′-GAGCAACTTCTTTCAACCACAG-3′; *GAPDH* (FOR): 5′-GAAGGTGAAGGTCGGAGTC-3′; GAPDH (REV): 5′-GAAGATGGTGATGGGATTTC-3′. The PCR machine used for both cDNA synthesis and RT-PCR was the Labcycler PCR machine (SensoQuest, Göttingen, Germany).

### 4.4. Real-Time (Quantitative) PCR (qPCR)

QPCR reactions were performed using KAPA SYBR FAST qPCR Master Mix (2×) kit (Kapa Biosystems, Cape Town, South Africa), according to the manufacturer’s instructions. All primer sequences for performing qPCR analysis were designed to cross an intron-exon boundary. The primer sequences are mentioned in Section 4.3. cDNA (15–20 ng) was added into the master mix and loaded (10 μL/well) in triplicate in a 96-well microplate (AB-0700, Invitrogen, Waltham, MA, USA), which was sealed with a Microseal “B”seal (BioRad, Watford, UK). QPCR was carried on a CFX96 Real-Time System (96-well block) (BioRad, Hercules, CA, USA) using the CFX Maestro software (BioRad, Cat.: 12004110, Hercules, CA, USA). Relative quantification analysis was performed for all qPCR reactions after normalization to the housekeeping gene (*Glyceraldehyde-3-phosphate dehydrogenase*, *GAPDH*). For each assay a no template control (NTC) was included to rule out the possibility of contamination. Two independent experiments were conducted. Within each experiment, each treatment condition was repeated in duplicate. The qPCR plate was set up to include triplicates of each treatment condition. The mean ± SD plotted is derived from triplicate PCR samples.

### 4.5. Cell Viability Assays

Cell viability was quantified using the MTT (3-(4,5-Dimethylthiazol-2-yl)-2,5-Diphenyltetrazolium Bromide) assay (Invitrogen, Waltham, MA, USA). Cells were incubated with ΜΤΤ (1 mg/mL) for 4 h at 37 °C wrapped in foil. The MTT was removed from each well and the insoluble purple formazan product was dissolved in DMSO (100 μL) following by plate shaking for 20 min in the dark. The purple formazan product was measured at a wavelength of 570 nm using a plate reader (Perkin Elmer Wallac Victor 1420-002 Multilabel Counter, GMI, Ramsey, NJ, USA). Four technical replicates were included in all experimental set ups. Medium-only control was included in all experiments performed to be able to measure background absorbance, which was then subtracted from the treatment groups.

#### 4.5.1. Cell Viability of CC Cells in Response to DAC Treatment

HeLa, CaSki, C33A and HaCat cell lines were seeded in 96-well plates at 5 × 10^4^ cells/well for 24 h. Cells were then treated with DAC treatment at low and high doses as indicated (0.015 μΜ, 0.03 μΜ, 0.0625 μΜ, 0.125 μΜ, 0.25 μΜ, 0.5 μΜ, 1 μΜ, 2 μΜ, 4 μΜ, 8 μΜ, 16 μΜ, 32 μΜ) in a total volume of 100 μL for 72 h. Culture medium was removed, and fresh medium with DAC was added to the cells every 24 h. Cell viability was assessed after 72 h of treatment. PBS-treated cells were used as the vehicle control. Three independent experiments were conducted for the cell viability assays. Within each experiment, four technical replicates were included. The data presented are plotted as the mean ± SD of the four technical replicates. Cell viability was also assessed at later time points in response to DAC treatment as follows: HeLa and CaSki cells were treated with DAC (0.3 μΜ or 1 μΜ) for 72 h. DAC was removed 24 h following the last treatment and replaced with fresh drug-free medium. Cells were allowed to grow in drug-free conditions up to 11d post-treatment. Cell viability assay was performed at 0d, 3d, 5d, 7d and 11d after DAC removal. At least two independent experiments were conducted for the long-term cell viability assays. Within each experiment, four technical replicates were included. The data presented are plotted as the mean ± SD of the four technical replicates.

#### 4.5.2. Cell Viability of CC Cells in Response to Cisplatin Treatment

HeLa, CaSki and C33A cells were seeded in 96-well plate for 24 h. Following 24 h of seeding, cells were treated with low and high cisplatin doses, as indicated (0.1560 μΜ, 0.3125 μM, 0.6250 μΜ, 1.25 μΜ, 2.5 μΜ, 5 μΜ, 10 μΜ, 20 μΜ), for 72 h. Culture medium was removed and fresh medium containing cisplatin was added into the cells every 24 h. Following 72 h of treatment, culture medium was discarded, and cells were allowed to grow in drug-free medium up to 4 days post-treatment. Cisplatin stock was dissolved in DMSO, and further dilutions to the desired concentrations were performed in culture medium. DMSO-treated cells were used as the vehicle control for each of the corresponding cisplatin doses. Two independent experiments were conducted. Within each experiment, four technical replicates were included. The data presented are plotted as the mean ± SD of the four technical replicates.

#### 4.5.3. Cell Viability of CC Cells in Response to Combination Treatment

C33A and CaSki cells were seeded in 96-well plate for 24 h. Following 24 h of seeding, cells were pre-treated with a low dose of DAC at 0.3 μΜ for 72 h. Fresh-media-containing DAC was added to the cells every 24 h. Following 72 h of DAC treatment, cells were allowed to grow under drug-free conditions up to 4d post-treatment. At 4d post-treatment cisplatin was added at 600 nM for 72 h. Culture medium was then discarded, and fresh medium without drug was added to the cells. Cells were allowed to grow under drug-free conditions up to 4 days after cisplatin removal. Cells treated with DAC alone or cisplatin alone were also included. PBS-treated and DMSO-treated cells were used as controls. Three independent experiments were conducted. Within each experiment, four technical replicates were included. The data presented are plotted as the mean ± SD of the four technical replicates.

### 4.6. Cell Counting

HeLa cells were seeded in 12-well plates for 24 h. Following 24 h of seeding, cells were treated with low-dose DAC treatment at 0.3. μΜ for 72 h. Cells were treated with fresh-media-containing DAC every 24 h. Following 72 h of DAC treatment, cells were allowed to grow under drug-free conditions up to 4d post-treatment. Cells then received cisplatin at 1.8 μΜ or 3.6 μΜ. Fresh-media-containing cisplatin was added to the cells at 24 h, 48 h and 72 h. Changes in cell numbers in response to combination treatment were monitored using the TC20 automated cell counter (Bio-Rad, Hercules, CA, USA). Measurements were taken at 24 h, 48 h and 72 h of cisplatin treatment and at 4d after cisplatin treatment. Cells treated with DAC alone (0.3 μΜ) and cisplatin alone (1.8 μΜ or 3.6 μΜ) were included in the experiment. Drug dilutions were prepared using PBS and DMSO for normalization purposes. Cells treated with PBS and DMSO without drugs were used as the vehicle control. Following completion of treatments, the medium from each well was removed and was used for counting the non-adherent cells. Adherent cells were washed with 1x PBS. Cells were then trypsinized, centrifuged at 1400 rpm for 5 min and were then resuspended in medium. For the cell counting, 10 μL of the cell suspension was loaded onto a slide, which was then inserted into the TC20 cell counter. Three independent experiments were conducted. Within each experiment, four technical replicates were included. The data presented are plotted as the median with range of the four technical replicates.

### 4.7. Immunocytochemistry (ICC)

HeLa, CaSki, C33A and HaCat cells were seeded on sterile coverslips in six-well plates at pre-optimized cell densities. ICC was performed to assess the levels of dsRNA in response to DAC treatment (1 μΜ, 72 h) in CC cell lines at the indicated time points (0d, 3d, 5d, 7d, 11d post-treatment). The ICC procedure was performed as described before [20]. Cells were treated with low-dose DAC treatment at 0.3 μΜ, 72 h. Culture medium was discarded and replaced with fresh medium containing DAC every 24 h. Cells were allowed to grow under drug-free conditions up to the end points of the experiment. Culture medium was removed, and cells were washed with 1× PBS. Cell fixation was followed using cold methanol (100%) at −20 °C for 15 min. Cells were then washed three times with ice-cold 1× PBS and a blocking step using 1% bovine serum albumin (BSA) prepared in 1× PBS was performed for 1 h at RT. Cells were then incubated with the primary antibody, mouse monoclonal J2 anti-dsRNA IgG2a antibody (1:50) (Scicons, Szirak, Hungary) overnight at 4 °C. Cells were washed three times with PBST for 10 min at RT followed by incubation with the secondary antibody, mouse IgG Kappa binding protein (m-IgGκ BP)-HRP (1:300) (Santa Cruz, TX, USA) for 1 h at RT. Following incubation, cells were washed three times with PBST for 10 min at RT. Nuclear staining was then followed by incubating cells with Hoechst 33342 (Invitrogen, Waltham, MA, USA) for 5 min at RT in the dark. Cells were then washed two times with PBST for 5 min. Mounting was then performed by adding 50 μL Prolong Gold antifade reagent with DAPI (Invitrogen, Waltham, MA, USA) on a microscope slide and then inverting the coverslips carefully on top of the antifade reagent ensuring that no air is trapped. Prior to imaging, the coverslips were sealed with nail vanish. Cell imaging was performed using the ZEISS Axio Observer.A1 microscope (Oberkochen, Germany). The exposure time applied for each time-point and condition was adjusted based on the lowest exposure time used for the sample that highly expressed the protein of interest. Protein quantification was performed using ImageJ software (Science Tech Blog, 2011, Version 1.8.0) followed by measuring the corrected total cell fluorescence (CTCF) as follows: CTCF = Integrated Density − (Area of selected cell × Mean fluorescence of background readings), as previously described [20]. The CTCF was calculated from 100 cells for each treatment condition and plotted as the median with range. Within each experiment, treatment conditions were repeated in duplicates. Two independent experiments were conducted.

### 4.8. Cell Cycle Analysis

Cells were seeded in six-well plates and treated with DAC at 0.3 μΜ and 1 μΜ for 72 h. DAC treatment was performed as described in Section 4.2. Cell cycle analysis using flow cytometry was examined at 0d, 3d, 5d, 7d and 11d post-treatment. Following DAC treatment, cells were washed once with sterile 1× PBS and trypsinized using 0.05% trypsin-EDTA (1×) (Gibco, Invitrogen, Garlsbad, CA, USA) for 5 min at 37 °C. Centrifugation at 1400 rpm for 5 min was performed, and the supernatant was discarded. The cell pellet was resuspended and fixed with 70% ice-cold ethanol for 2 hrs at 4 °C. Cell suspension was centrifuged, and the cell pellet was washed once with ice-cold 1× PBS. Cells were pelleted by centrifugation at 1400 rpm for 5 min at 4 °C. The supernatant was discarded, and the cell pellet was resuspended in 1×PBS with 0.2 mg/mL RNase A (Macherey-Nagel, Fisher Scientific, Loughborough, UK) and 0.02 mg/mL Propidium iodide (PI) (Invitrogen, Eugene, OR, USA) in a total volume of 500 μL. Following incubation of cells for 30–45 min at 37 °C, cells were passed through the FACS filter tubes (Corning, Glendale, AZ, USA). Cell cycle analysis was performed using the S3e Cell Sorter machine (Bio-Rad, RRID: SCR_019710, Hercules, CA, USA). Cells that were not stained with PI were used as the no-staining control. Untreated and PBS-treated cells (vehicle control) were used as controls. Data analysis was performed using the FlowJo V10 software (Version 10.6.2, RRID: SCR_008520, Ashland, OR, USA). The sub G1, G0/G1, S, G2/M phases were identified, and the percentage populations were plotted. Three independent experiments were conducted. Within each experiment, each treatment condition was repeated in duplicate.

### 4.9. Fluorescence-Activated Cell Sorting (FACS)

HeLa, CaSki and C33A cells were seeded in six-well plates and treated with DAC at 1 μΜ for 72 h as described above (Section 4.2). FACS analysis was performed at 3d and 11d post-treatment. Cells were harvested by trypsinization and centrifuged at 1400 rpm for 5 min. The supernatant was discarded, and the cell pellet was washed with ice-cold PBS followed by centrifugation at 1400 rpm for 5 min at 4 °C. The supernatant was discarded, and the cell pellet was resuspended in antibody solution (1:100). The cells were stained with anti-MHC-class I (PE mouse IgG2a, BioLegend, Amsterdam, The Netherlands), Annexin V/7-AAD (used Annexin V binding buffer) (Pacific Blue, BioLegend, Amsterdam, The Netherlands) or anti-PDL1 (CD274, PE-Cyanine 7, eBioscience, Waltham, MA USA) for 45 min at 4 °C. Cells were then washed with ice-cold 1× PBS and centrifuged with 1400 rpm for 5 min at 4 °C. The supernatant was discarded, and cell pellet was resuspended in 500 μL ice-cold 1× PBS. For the cells stained with Annexin V/7AAD, the cells were incubated for 15 min at room temperature in the dark. Following incubation, 400 μL of Annexin V Binding Buffer was added to each tube followed by FACS analysis. The cell suspension was passed through the FACS filter tubes (Corning, Glendale, AZ, USA). For normalization purposes, CountBright absolute counting beads (Invitrogen, Eugene, OR, USA) were added at 1:100 into each FACS tube without passing the filter. Untreated and PBS-treated cells (vehicle control) were used as controls. Cells without staining were used as the background control. In addition, samples incubated with a mouse IgG1 Kappa Isotype Control (PE-Cyanine 7, eBioscience, Waltham, MA, USA) were used as a control. Data analysis was performed using FlowJo_V10 software (Version 10.6.2, RRID: SCR_008520, Ashland, OR, USA). Three independent experiments were conducted for each marker. Within each experiment, each treatment condition was repeated in duplicate.

### 4.10. Statistical Analysis

Statistical analysis and graphs were created using GraphPad software (Prism7, Version 8.0, RRID: SCR_002798, La Jolla, CA, USA). To assess which statistical test to select, we used D’Agostino–Pearson omnibus normality test. For normally distributed data or not normally distributed data, parametric (one-way ANOVA, Tukey’s multiple comparisons test) and non-parametric tests (one-way ANOVA with Kruskal–Wallis test, Dunn’s multiple comparisons post hoc test or two-tailed Mann–Whitney *t*-test) were applied respectively, where indicated. Statistically significant differences were defined as follows: * *p* < 0.05, ** *p* < 0.01, *** *p* < 0.001, **** *p* < 0.0001. The alpha value was set at 0.05.

## 5. Conclusions

In conclusion, this study highlights the anti-cancer, immunomodulating and chemosensitizing potential of low-dose DAC treatment in CC cell lines in vitro. These effects were determined using end-point in vitro assays developed in our study. Our data showed that DAC treatment resulted in a potent activation of a viral mimicry response, as indicated by the induction of cytoplasmic dsRNA and downstream activation of *MDA5* and *IRF7*. The induction of an anti-viral immune response by DAC treatment in CC cells may pave the way to novel combinatorial therapeutic approaches. Our data also showed that DAC treatment significantly reduces cell viability, induces G2/M phase cell cycle arrest and apoptosis and increases response to chemotherapy. Furthermore, we showed a prolonged upregulation of MHC-class I and PD-L1 in CC cells in vitro. Overall, the immunoregulating capacity of DAC could increase tumour visibility to immune surveillance by the host and promote tumour immunogenicity, further enhancing its therapeutic potential in CC. However, further studies are needed in order to verify whether DAC-mediated changes in MHC-I and PD-L1 in CC cells are clinically significant. The effects of DAC treatment were tested in both HPV+ and HPV− cells showing that the anti-cancer and immunomodulating potential of DAC treatment is independent of the HPV status of the cells. Our data indicate the promising anti-cancer effect of DAC in other cancer types that are not HPV-associated cancers. This study provides evidence indicating the potential of using low dose DAC as an immunomodulator and/or chemosensitizer in CC, further enhancing the therapeutic management of CC patients and paving the way to new therapeutic approaches.

## Data Availability

The data of this study can be provided upon request by the corresponding author.

## Supplementary Materials

The following supporting information can be downloaded at: https://www.mdpi.com/article/10.3390/ijms232214042/s1.
