# Supplementary material for: Decitabine Treatment Induces a Viral Mimicry Response in Cervical Cancer Cells and Further Sensitizes Cells to Chemotherapy
Source: Int J Mol Sci. 2022 Nov 14;23(22):14042. doi: 10.3390/ijms232214042 (PMC9692951; doi:10.3390/ijms232214042)
Supplement: Supplementary file 1 [file ijms-23-14042-s001.zip › ijms-1888093-supplementary.pdf]

Supplementary Materials

The following supporting information are listed:

Figure S1. Cell viability of CC cell lines in response to DAC treatment;

Figure S2: A timeline of the endpoint assays for each timepoint post-treatment in CC cells;

Figure S3: DAC treatment induces dsRNA levels in CC cells and in normal immortal keratinocytes;

Figure S4: Changes in cell viability of CC cells in response to low and high cisplatin doses;

Figure S5: Low dose DAC treatment followed by cisplatin treatment increase the number of non-adherent HeLa cells in the supernatant.

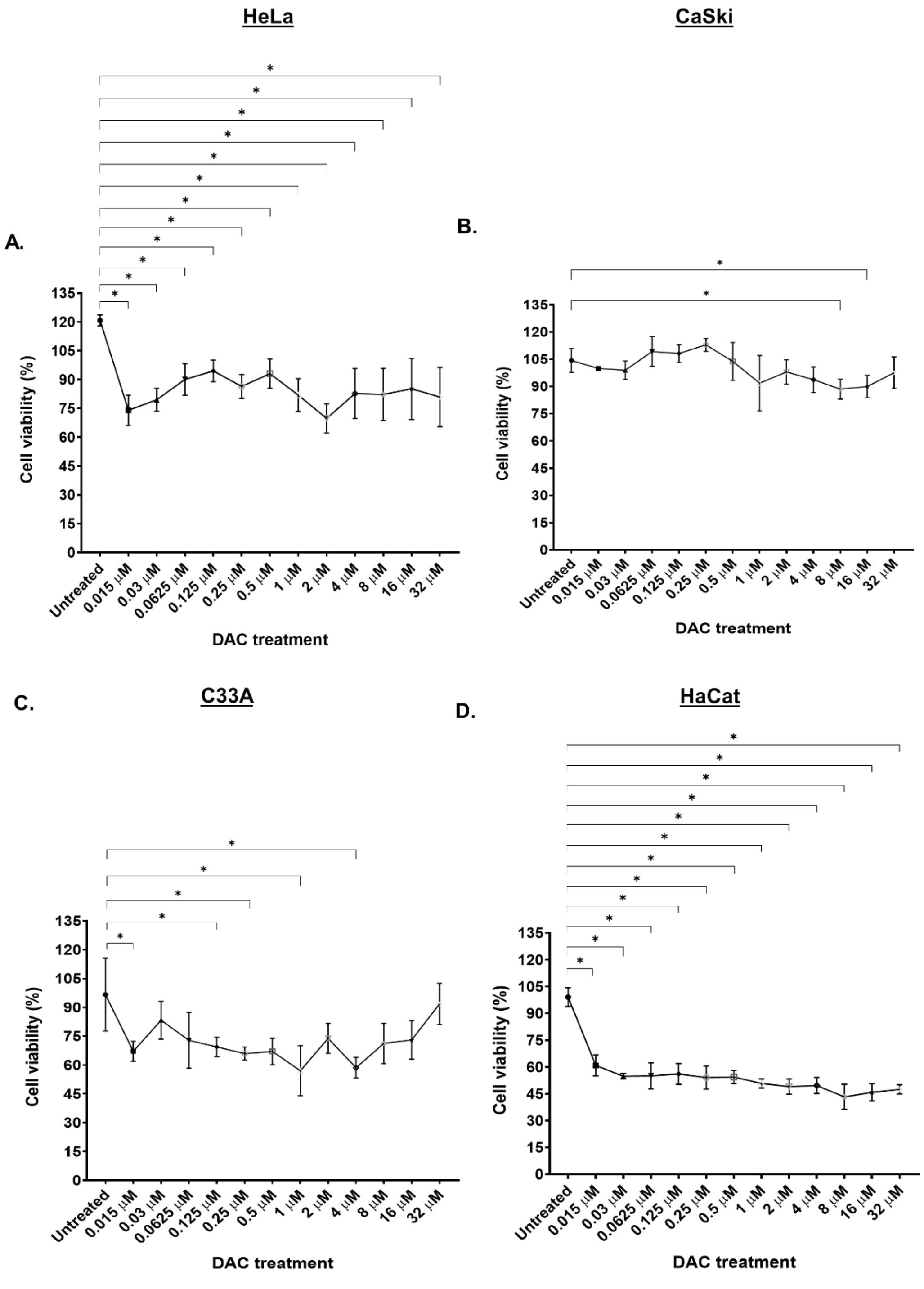

Figure S1. Cell viability of CC cell lines in response to DAC treatment. Cell viability analysis of CC cell lines in response to 72 h DAC treatment at different doses (32 µM, 16 µM, 8 µM, 4 µM, 2 µM, 1 µM, 0.5 µM, 0.25 µM, 0.125 µM, 0.0625 µM, 0.03 µM, 0.015 µM) using MTT assay. The graphs show the cell viability (%) normalized to the vehicle control (PBS) for HeLa (**A**), CaSki (**B**), C33A (**C**) CC cell lines and HaCat immortal keratinocyte cell line (**D**). The data is a representative repeat of three independent experiments. The data is plotted as the mean ± SD. Statistical analysis was performed using Mann Whitney *t*-test (* $p < 0.05$).

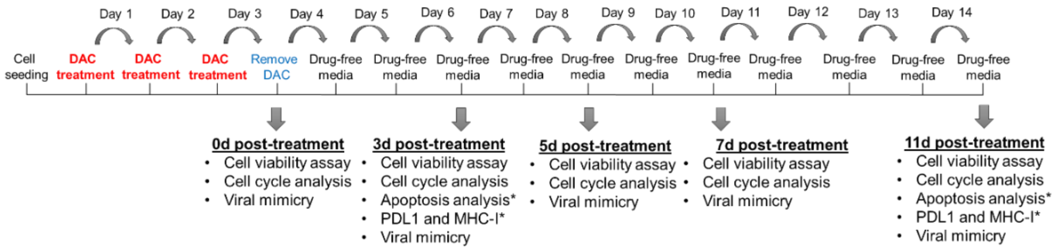

Figure S2: A timeline of the endpoint assays for each timepoint post-treatment in CC cells. Cells were treated with DAC for 72 h. Following 24 h of DAC treatment completion, cells were allowed to grow under drug-free conditions. Endpoint assays were developed to assess the anti-cancer, immunomodulating and chemosensitizing potential of DAC treatment in CC cell lines at 0 days, 3 days, 5 days, 7 days and 11 days post-treatment. These include assessing induction of viral mimicry response, cell viability assay, cell cycle analysis, apoptosis, induction of immune-related genes. * For these parameters two time points were selected (3d and 11d post-treatment) to compare between the early and late timepoints after DAC removal.

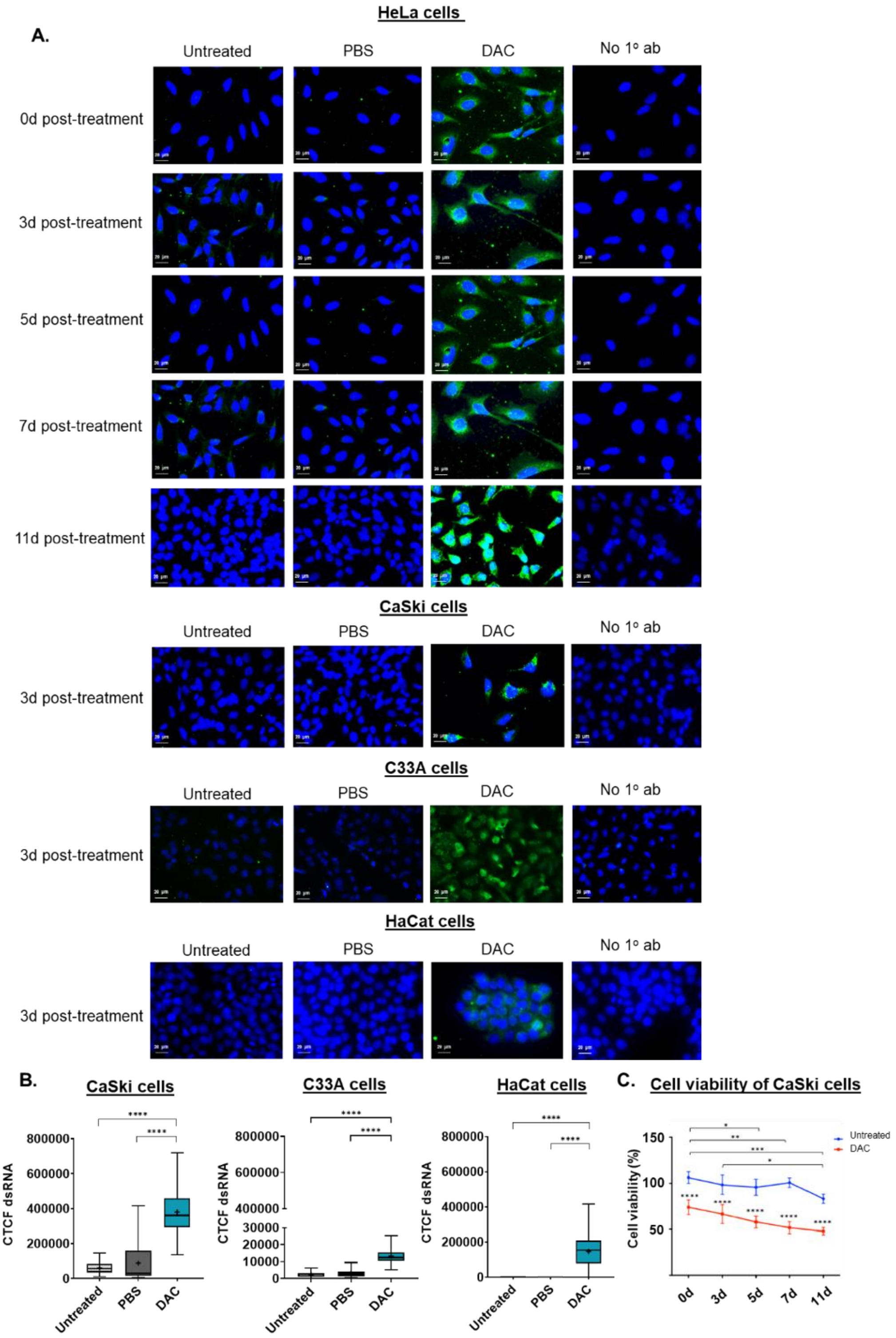

Figure S3: DAC treatment induces dsRNA levels in CC cells and in normal immortal keratinocytes. (**A**) ICC/IF analysis of dsRNA following DAC treatment (1 μM, 72 h) in HeLa, CaSki, C33A cells and HaCat keratinocyte cell line at the indicated time points. (**B**) CTCF was calculated for CaSki, C33A and HaCat cells using ImageJ as previously described [20]. Data represents the median with range of CTCF value from 100 cells per treatment condition. The mean is also indicated by a cross (+). The experiment is a representative repeat of two independent experiments. Statistical analysis was conducted using One-way ANOVA (Kruskal-Wallis test) (**** $p < 0.0001$). (**C**) Cell viability of CaSki cells in response to DAC treatment (1 μM) for 72 h at 0d, 3d, 5d, 7d and 11d post-treatment using an MTT assay. The data presented is a representative repeat of two independent experiments. The data is plotted as the mean ± SD. Statistical analysis was conducted using Two-way ANOVA with Tukey's multiple comparisons test (* $p < 0.05$, ** $p < 0.01$, *** $p < 0.001$, **** $p < 0.0001$).

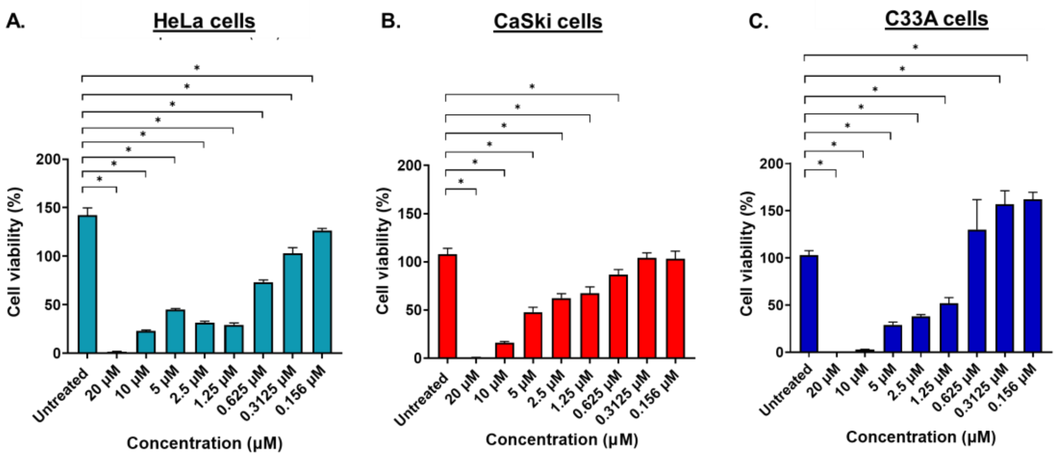

Figure S4: Changes in cell viability of CC cells in response to low and high cisplatin doses. Cell viability analysis of HeLa (**A**), CaSki (**B**) and C33A cells (**C**) in response to cisplatin treatment alone (72 h) at the indicated concentrations using MTT assay. The MTT assay was performed at 4d post-treatment. The data presented is a representative repeat of two independent experiments. The data is plotted as the mean ± SD. Normalization to the vehicle control (DMSO-treated cells) was conducted. Statistical analysis was performed using Mann-Whitney *t*-test (* $p < 0.05$).

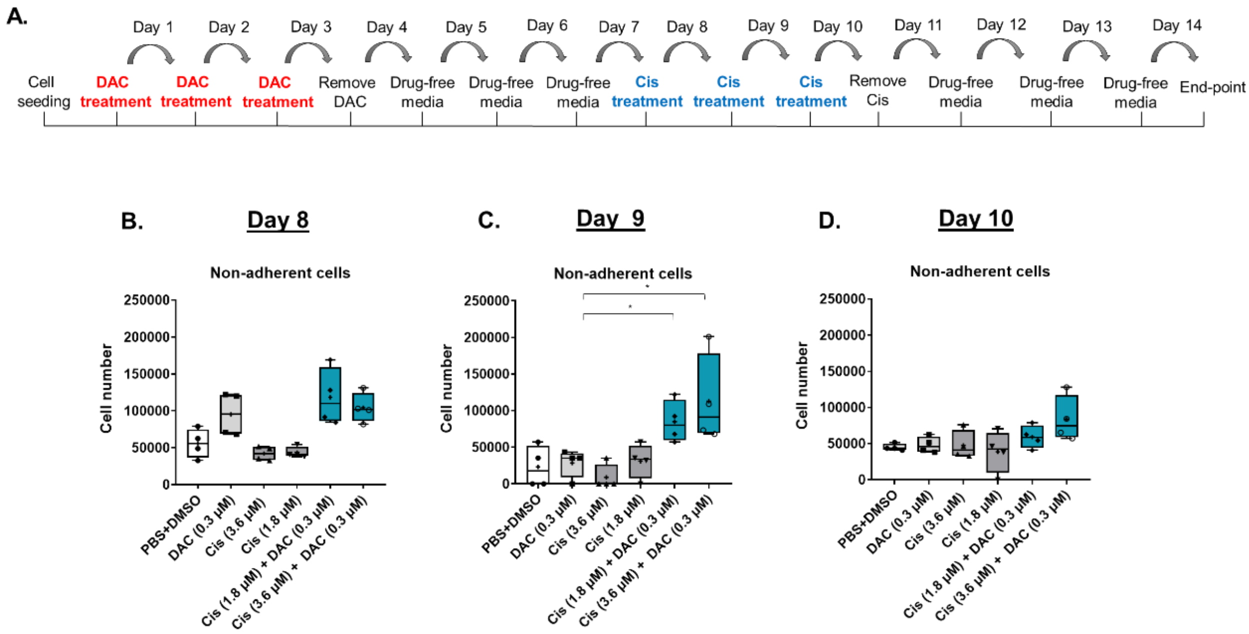

Figure S5: Low dose DAC treatment followed by cisplatin treatment increase the number of non-adherent HeLa cells in the supernatant. (**A**) Combination treatment timeline of HeLa cells treated with low dose DAC (0.3 μM, 72 h) followed by cisplatin treatment (1.8 μM or 3.6 μM, 72 h). Absolute cell number of non-adherent cells in response to DAC alone, cisplatin alone or combination treatment at the indicated time points: Day 8 (**B**), Day 9 (**C**) and Day 10 (**D**). Cell counting was performed using an automatic cell counter. The data presented is a representative repeat of three independent experiments. The graphs show the median with range of the counted cell numbers. The mean is also indicated by a cross (+). Statistical analysis was performed using One-way ANOVA (Kruskal-Wallis test) test (* $p < 0.05$).
